# The Effects of Low Doses of Two *Fusarium* Toxins, Zearalenone and Deoxynivalenol, on the Pig Jejunum. A Light and Electron Microscopic Study

**DOI:** 10.3390/toxins7114684

**Published:** 2015-11-11

**Authors:** Barbara Przybylska-Gornowicz, Michał Tarasiuk, Bogdan Lewczuk, Magdalena Prusik, Natalia Ziółkowska, Łukasz Zielonka, Maciej Gajęcki, Magdalena Gajęcka

**Affiliations:** 1 Department of Histology and Embryology, Faculty of Veterinary Medicine, University of Warmia and Mazury in Olsztyn, Oczapowskiego 13, 10-718 Olsztyn, Poland; E-Mails: przybylb@gmail.com (B.P.-G.); lewczukb@uwm.edu.pl (B.L.); mprusik@gmail.com (M.P.); ntrzaska@o2.pl (N.Z.); 2 BIOMIN Polska Sp. z o.o., Grochowska 16, 04-217 Warszawa, Poland; E-Mail: michal.tarasiuk@biomn.net; 3 Department of Veterinary Prevention and Feed Hygiene, Faculty of Veterinary Medicine, University of Warmia and Mazury in Olsztyn, Oczapowskiego 13, 10-718 Olsztyn, Poland; E-Mails: lukaszz@uwm.edu.pl (L.Z.); gajecki@uwm.edu.pl (M.G.)

**Keywords:** mycotoxins, zearalenone, deoxynivalenol, histology, ultrastructure, jejunum, pig

## Abstract

Immature gilts were administered *per os* with zearalenone (ZEN) at 40 μg/kg BW (group Z, *n* = 9), deoxynivalenol (DON) at 12 μg/kg BW (group D, *n* = 9), a mixture of ZEN and DON (group M, *n* = 9) or a placebo (group C, *n* = 9) over a period of six weeks. The pigs were sacrificed after one, three, or six weeks of the treatment (12 pigs per each time-point). Histological investigations revealed an increase in the mucosal thickness and the crypt depth as well as a decrease in the ratio of the villus height to the crypt depth in groups D and M after six weeks of exposure to the mycotoxins. The number of goblet cells in the villus epithelium was elevated in groups Z and M after one week and in group D after three weeks. The administration of ZEN increased the lymphocyte number in the villus epithelium after 1 week and the plasma cell quantity in the lamina propria after one, three, and six weeks of the experiment. DON treatment resulted in an increase in the lymphocyte number in the villus epithelium and the lamina propria after six weeks, and in the plasma cell quantity in the lamina propria after one, three, and six weeks of exposure. In group M, lymphocyte counts in the epithelium and the lamina propria increased significantly after six weeks. Neither mycotoxin induced significant adverse changes in the ultrastructure of the mucosal epithelium and the lamina propria or in the intestinal barrier permeability. Our results indicate that immune cells are the principal target of low doses of ZEN and DON.

## 1. Introduction

Zearalenone (ZEN) and deoxynivalenol (DON) are *Fusarium* mycotoxins that have been extensively studied because of their wide distribution in nature and their ability to cause pathological changes in animals and humans. Pigs are a good model for the investigation of various aspects of the toxic effects of ZEN and DON as they are highly sensitive to these compounds and the porcine digestive tract is similar to the human gastrointestinal system [1,2,3,4,5]. The digestive system, in which the mycotoxins are absorbed [6,7], metabolized [7,8,9,10], and broken down [6], is extensively exposed to the toxic effects of these substances and their metabolites [2,4,11,12,13].

Several mechanisms have been proposed for biological effects of DON [11,12,13,14,15,16,17,18,19]. This mycotoxin acts as protein synthesis inhibitor [14,15] and, as a consequence, decreases claudin and occludin expression in epithelial cells and weakens the intestinal barrier, increasing its permeability to bacteria [12,13]. DON also simulates mitogen-activated protein kinases, which cause a wide variety of biological effects [11,19]. Moreover, the mycotoxin induces a proinflammatory response by the stimulation of cytokine formation [11,16,17]. Cytokines seem to be responsible for many of the negative effects of DON [11,16]. Anorexia caused by DON is a consequence of changes in various regulatory mechanisms, including the secretion of serotonin and the gut peptide YY [11,18]. Lowest observed effect levels (LOEL) of DON that induced vomiting in pigs range from 50 to 100 μg/kg BW, while no observed adverse effect level (NOEL) of this toxin proposed for pigs-from 25 to 75 μg/kg BW [20].

ZEN and its metabolites have estrogenic activity and compete with endogenous hormones for the binding sites of the estrogen receptors [1,21]. Moreover, ZEN affects the activities of the enzymes involved in steroid metabolism: 3-β-hydroxysteroid dehydrogenase type 1, cytochrome P450 side-chain cleavage enzyme, and P450 aromatase [1]. Treatment with ZEN results in hyperestrogenism, precocious puberty, and reproductive disorders [21]. Exposure of sows to ZEN during pregnancy and lactation reduces the quantity of healthy follicles in piglets in the F1 generation, which might lead to premature oocyte depletion in adulthood [22]. ZEN and its metabolites also have deleterious effects on immune functions [23]. LOEL proposed for the oestrogenic effect of ZEN in mature and immature female pigs ranges from 17 to 200 μg/kg BW per day [24]. The JECFA established NOEL for pigs on the basis of oestrogenic effects at the level of 40 μg/kg BW per day [25].

In many cases, a diet contains DON and ZEN simultaneously [26], however the effects of concurrent exposition to these mycotoxins are poorly recognized and difficult to predict [1,11].

Most *Fusarium* mycotoxins are absorbed in the proximal fragment of the small intestine [27,28,29,30], which suggests that the jejunum plays an important role in the process of ZEN and DON absorption and therefore is particularly prone to a direct effect from these compounds. The *Fusarium* mycotoxins are also partially metabolized in the jejunum [31]. Studies investigating the concentrations of mycotoxins and their metabolites in the intestinal wall in relation to the duration of exposure showed the highest levels of DON in the jejunum after three to four weeks of administration [32] and the highest concentrations of ZEN and its metabolites two to three weeks of exposure [33]. The results of studies concerning the influence of ZEN and DON on intestinal function demonstrated that the severity of changes in jejunal morphology is determined by the dose and duration of exposure to the mycotoxins [2,4,11,16]. According to previous studies, the mycotoxins can contribute to (i) changes in intestinal barrier function [11,12,13,16]; (ii) changes in goblet cell activity [11]; and/or (iii) changes in the activity of the local immune system [2,11,17,34].

The worldwide reported high rate of occurrence of mycotoxins at low concentrations in food and feed [26] leads to the conclusion that both humans and animals are frequently exposed to low doses of various mycotoxins for a shorter or longer period. These doses are usually too low to induce directly occurring clinical symptoms or even specific manifestations of chronic intoxication, however the intake of low doses of mycotoxins may result in more or less serious damages of cells, tissues, and organs. Therefore, it is very important to study the influence of such doses of mycotoxins, given alone or in combination, on the organisms.

The objective of this study was to determine the effect of low doses of ZEN and DON, administered *per os* for one, three, or six weeks, individually or together, on the histology and ultrastructure of the jejunum in pre-pubertal gilts. In addition, the effect of the mycotoxins on the permeability of the intestinal epithelial barrier was studied using the lanthanum method.

The dose of DON used in our study, 12 µg/kg BW, is much lower than the NOAEL proposed for pigs [20]. In comparison, the total human intake of DON according to the Global Environment Monitoring System ranges from 0.77 μg/kg BW per day in the African diet to 2.4 μg/kg BW per day in the Middle Eastern diet [35]. The dose of ZEN we used, 40 µg/kg BW, was the same as the NOAEL established by JECFA for pigs [25]; however, it should be noted that this value was proposed for the estrogenic effects, while our study was concerned with the effects on the intestine. The European Commission decided to establish the limit of DON content in feed (for complementary and complete feedstuffs) at 0.9–5 mg/kg, and the limit of ZEN at 0.1–0.5 mg/kg, depending on the species [36].

## 2. Results and Discussion

### 2.1. Light Microscopic Study

#### 2.1.1. Architecture of the Mucosa

**Figure 1:**
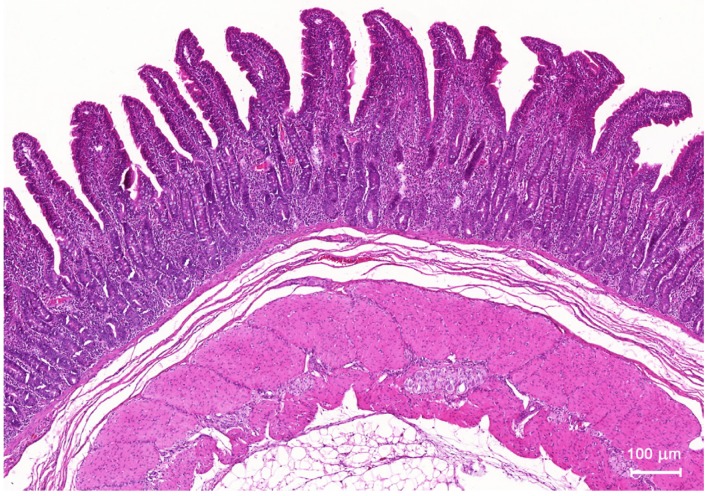
Histological section of the jejunal wall of a control pig sacrificed at the end of the experiment. HE staining.

The jejunum of the pre-pubertal gilts was characterized by well-developed, finger-shaped villi and numerous intestinal crypts inside the lamina propria (Figure 1). The mucosa was covered by a columnar epithelium comprising primarily absorptive enterocytes and goblet cells. Lymphocytes were present between the epithelial cells. The interior of the villi and the spaces between the crypts were filled with loose connective tissue containing numerous blood vessels and cells of the immune system—lymphocytes and plasma cells. The well-developed muscularis mucosa separated the mucosa from the tunica submucosa, which had a characteristic loose arrangement. The muscularis and the serosa also had a regular structure. No significant qualitative differences in the intestinal architecture were observed between the groups of animals investigated; however, the mycotoxins influenced the quantitative parameters of the mucosa.

**Figure 2:**
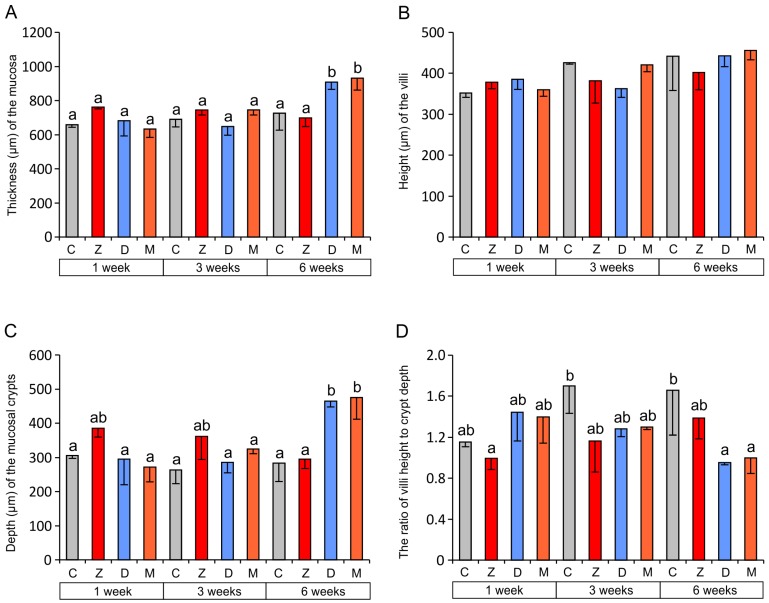
Morphometric characteristic of the jejunal mucosa. (**A**) Thickness of the mucosa; (**B**) Height of the villi; (**C**) Depth of the mucosal crypts; (**D**) The ratio of villus height to crypt depth. Values presented are means and SEM. The capital letters under horizontal axis: C—control group, Z—group treated with ZEN, D—group treated with DON, M—group treated with ZEN and DON. Means annotated with different lower case letters above the bars are significantly different at *p* ≤ 0.05.

The administration of mycotoxins led to a significant increase in the mucosal thickness in the group receiving DON (D) and the group receiving DON + ZEN (M) compared to the control group (C) and the group receiving ZEN (Z) after six weeks of treatment (Figure 2A). No significant differences were noted after one and three weeks of treatment (Figure 2A). The villi and crypts constitute the structural and functional base for the small intestine function. A morphometric analysis of these mucosal structures revealed that only the crypts were affected by the mycotoxins (Figure 2B,C). A significant increase in the crypt depth was noted in groups D and M compared to the control group after six weeks of the toxins administration (Figure 2C). As a result of these changes, a significant decrease in the ratio of the villus height to the crypt depth was found in groups D and M after six weeks (Figure 2D).

It is noteworthy that the ZEN and DON doses we used had no significant effect on the qualitative and quantitative characteristics of the villi. Morphological changes in the villi are generally considered as a robust indicator of the intestinal response to dietary modifications, the harmful effects of anti-nutritional factors and toxins in feed, and the digestive pathology [37,38,39]. Previous studies indicated that high and intermediate doses of *Fusarium* mycotoxins significantly affected the villus morphology [2,11,19,31]. The administration of feed contaminated with DON (3 mg/kg) to five-week-old piglets over a period of five weeks led to atrophy, fusion, and shortening of the villi [2]. Similar results were also reported after administration of naturally contaminated feed containing DON at levels of 1.5–3.0 mg per 1 kg of feed [40,41]. A decrease in villus height was also noted after incubation of explants of the pig jejunum in the presence of DON at concentrations higher than 0.2 μM [3,12]. In contrast, the changes in the length and shape of the villi in the jejunum were not found after pigs were treated with ZEN at doses of 200 or 400 μg/kg BW for seven days [42]. Moreover, no changes in villus height were observed in the jejunum of pigs receiving feed naturally contaminated with DON (28.9 μg/kg of feed), T-2 toxin (11.5 μg/kg of feed) or ZEN (33.2 μg/kg of feed) for 14 days [43]. In other species, a decrease in villus height was reported in the duodenum of chickens and turkeys after administration of *Fusarium* mycotoxins, including DON [44,45]. Interestingly, diets containing DON, acetyl-DON, and ZEN induced opposite changes in the villus length in various segments of the chicken small intestine: a decrease in the duodenum and an increase in the jejunum and the ileum [46].

A significant decrease in the ratio of the villus height to the crypt depth found in our study after six weeks of DON administration alone or together with ZEN indicates changes in the function of the intestinal crypt-villus axis. A drop in this parameter of intestinal status is usually considered as a sign of insufficient cell proliferation in the growth zone in crypts or excessive exfoliation of cells in the villi [38,39,47]. It frequently results from apoptosis, induced by various factors, including feed contaminated with *Fusarium* mycotoxins [31,38,39,47]. In contrast to the above data, the changes in the ratio of villus height to crypt depth found in our study arose from hypertrophy of the crypts because no changes in the villus height were found. The mechanism of this phenomenon is unknown, but an increased cell proliferation inside the crypts and the remodeling of the crypts due to interaction with the connective tissue of the lamina propria should be considered [4,38]. Recently, an increase in crypt depth was reported in the jejunum of pigs receiving DON at the level of 0.9 mg/kg of feed [48] that is recommended as acceptable for complementary and complete feedstuffs for this species by the European Commission [36]. The changes in the crypt depth coincided with a decrease in the villus height [48].

The occurrence of the quantitative changes in mucosa after six weeks of DON and DON + ZEN administration, observed in our study, may be attributed to the progressive accumulation of mycotoxins in the intestinal wall to a concentration that affects the mucosa [32].

#### 2.1.2. Goblet Cells

**Figure 3:**
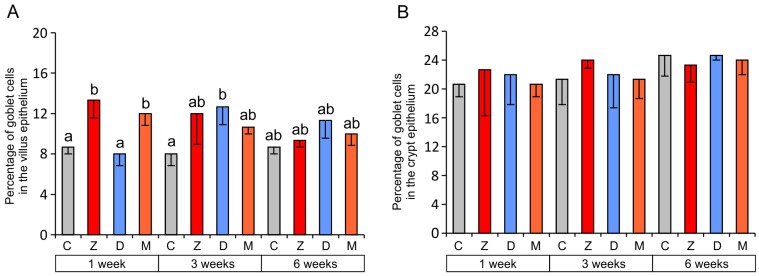
The percentage of goblet cells in the villus epithelium (**A**) and the crypt epithelium (**B**) of the jejunum. Values presented are the mean and SEM. For explanations, see Figure 2.

A significant increase in the number of goblet cells was noted in the epithelium lining jejunal villi in groups Z and M after one week of mycotoxin administration. The number of goblet cells in the villus epithelium was also significantly increased in group D at week three (Figure 3A). No significant changes were noted in the percentage of goblet cells in the crypt epithelium (Figure 3B).

Other authors’ findings indicate that mycotoxins influence goblet cells in the intestinal epithelium, but the results obtained vary considerably between studies and are inconclusive [2,11,40,42,43]. Obremski and co-workers [42] observed an increase in the number of goblet cells in the small intestine after the administration of ZEN at doses of 200 and 400 μg/kg BW for seven days. Conversely, fewer goblet cells were found in the jejunum of pigs after administration of a diet containing DON (28.9 μg/kg of feed), T-2 toxin (11.5 μg/kg of feed), and ZEN (33.2 μg/kg of feed) for 14 days [43]. Bracarnese *et al.* [2] and Gerez *et al.* [40] also reported fewer goblet cells in the jejunum after DON administration at 1.5–3 mg/kg feed for four to five weeks.

This study shows that changes in the relative number of goblet cells are transient and the time of their occurrence depends on the toxin administered. The increase in the percentage of goblet cells induced by ZEN was short-lived, and it was noted only in the initial period of the exposure. The changes caused by DON occurred after three weeks of administration and faded thereafter. Our data partially explain the discrepancies reported in the literature, indicating the duration of the administration of the mycotoxin as a source of the differences. An increase in the number of goblet cells is indicative of intensified mucus secretion and is most likely a protective mechanism against mycotoxins.

#### 2.1.3. Cells of the Immune System

**Figure 4:**
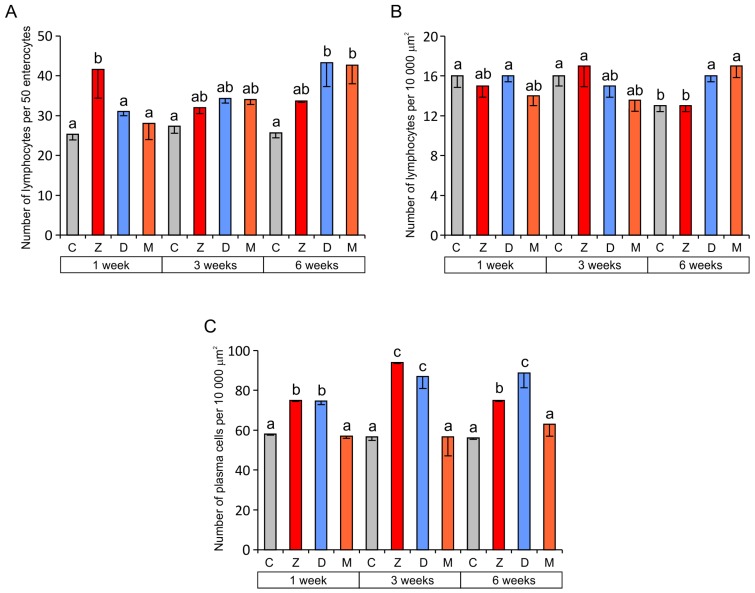
Immune system cells in the jejunum. (**A**) Number of lymphocytes in the villus epithelium (per 50 epithelial cells); (**B**) Number of lymphocytes in the lamina propria (per 10,000 µm^2^); (**C**) Number of plasma cells in the lamina propria (per 10,000 µm^2^). Values presented are the mean and SEM. For explanations, see Figure 2.

The results of quantitative analyses revealed a significant increase in the relative number of lymphocytes in the epithelium covering villi of the jejunum in group Z after one week and in groups D and M after six weeks of exposure compared to group C (Figure 4A). In the lamina propria, significantly higher counts of lymphocytes were found in groups D and M than in the control group after six weeks of exposure (Figure 4B).

The mechanisms of ZEN action on intestinal immune functions are largely unknown. One possible mechanism might be a direct effect of ZEN on lymphocytes and macrophages because estrogen receptors are present in these cells [23,49]. In previous studies, an increased occurrence of lymphocytes was found in the pig jejunum after ingestion of diets containing ZEN at doses of 200 and 400 μg/kg BW for seven days [42] or containing ZEN (33.2 μg/kg of feed), DON (28.9 μg/kg of feed), and T-2 toxin (11.5 μg/kg of feed) for 14 days [43]. However, it was demonstrated that the exposure of pigs to low doses of ZEN for several weeks intensified apoptosis and weakened proliferation of ileal Peyer’s patch lymphocytes [50]. The data cited agree with our results that show only a short-term increase in the relative number of epithelial lymphocytes after ZEN treatment.

The mechanism of the DON-induced increase in the number of lymphocytes in the epithelium and the lamina propria is most likely related to the direct activation of cytokine synthesis by this mycotoxin [11,17,51,52]. In mice, DON evoked upregulation of mRNAs encoding several proinflammatory cytokines including IL-1α, IL-1β, IL-6, IL-11, TNF-α, and TGF-β as well as T cell cytokines: IFN-γ and IL-2 [17]. DON (2.8 mg/kg feed) induced a significant increase in the expression of IL-1β, IL-2, IL-6, IL-12p40, and MIP-1β in the jejunum and TNF-α, IL-1β and IL-6 in the ileum [2]. Moreover, the increase in the expression of IL-1β, IL-8, MCP1, and IL-6 in pig intestinal loops exposed to *Salmonella* Typhimurium was potentiated when DON (1 µg/mL) was co-exposed with the bacteria [52]. DON (10 μM) also induced a pro-inflammatory response with a significant increase in expression of mRNA encoding for IL-8, IL-1α, and IL-1β, TNF-α in non-transformed intestinal porcine epithelial cells IPEC-1 and porcine jejunal explants [51]. The increased permeability of the epithelial barrier to antigens present in the intestinal lumen caused by a decrease in claudin and occludin production [2,11,13] should also be considered as a factor that induces lymphocyte proliferation. As demonstrated *in vitro*, DON (0.5–1 µg/mL) makes the intestinal epithelium more permeable to *Salmonella* Typhimurium [52]. Recent investigations showed that DON affects the composition of the basement membrane proteins and influences the migration of lymphocytes into the mucosal epithelium [53]. Zielonka *et al.* [54] revealed an increase in the proliferative activity of immunocompetent cells harvested from the jejunal and ileal lymph nodes of pre-pubertal, female wild boars exposed to DON (1008 μg/kg of feed) and ZEN (26 μg/kg of feed) for seven days in naturally contaminated feed. Therefore, it can be assumed that long-term (six-week) exposure to low and combined doses of ZEN and DON in feed intensifies lymphocyte proliferation in the mesenteric lymph nodes of the jejunum. According to Maresca [31] and Maresca & Fantini [34], such a process could increase the lymphocyte counts in the porcine jejunal epithelium. The opposite effect—a decreased infiltration of the pig jejunum by lymphocytes—was observed after the administration of DON at high doses, amounting to 1.5–3 mg/kg of feed for four to five weeks [2,40].

Quantitative analysis revealed that, regardless of the duration of mycotoxin administration, the number of plasma cells in the lamina propria was significantly higher in groups Z and D than in groups C and M (Figure 4C). Our results indicate that both ZEN and DON have a stimulatory effect on the immune system when given alone. However, the simultaneous application of both mycotoxins (group M) had no effect on plasma cells. This result is difficult to interpret. It could suggest that *Fusarium* mycotoxins have an antagonistic effect on plasma cells when administered in combination. An increase in the number of plasma cells is usually closely correlated with infiltration of the lamina propria by B cells from the mesenteric lymph nodes. However, such a simple relationship was not found in our study; therefore, it is possible that mycotoxins affect the process of transformation of B cells into plasma cells. Literature data partially explain the action of ZEN on plasma cells. It has been demonstrated that long-term treatment with low doses of ZEN (8 µg/kg BW for 14, 28, 42 days) decreases the expression of CD 21 on B cells and increases the percentage of the B1 cell subpopulation [55]. ZEN at the same dose also inhibits IL-2 and IFN-γ secretion and stimulates IL-4 and IL-10 production by Th lymphocytes [56]. Consequently, ZEN can promote formation of plasma cells and antibody production, leading to inflammation and allergy [34,56]. On the other hand, ZEN can promote lower resistance to viruses and tumors [56].

### 2.2. Ultrastructure of the Mucosa

Morphological changes in cells and tissues induced by toxins of natural origin and synthetic toxins start at the level of biological membranes and organelles, where they can be observed within minutes and hours after exposure. Then, depending on the toxin dose and the duration of its action, fine subcellular changes may lead to more pronounced cell damages, which are visible in histological analyses and later in anatomopathological examination. The morphological effects of low toxin doses might be restricted to fine structural changes. Therefore, we decided to perform ultrastructural studies of the samples taken after one, three, and six weeks after toxin administration.

**Figure 5:**
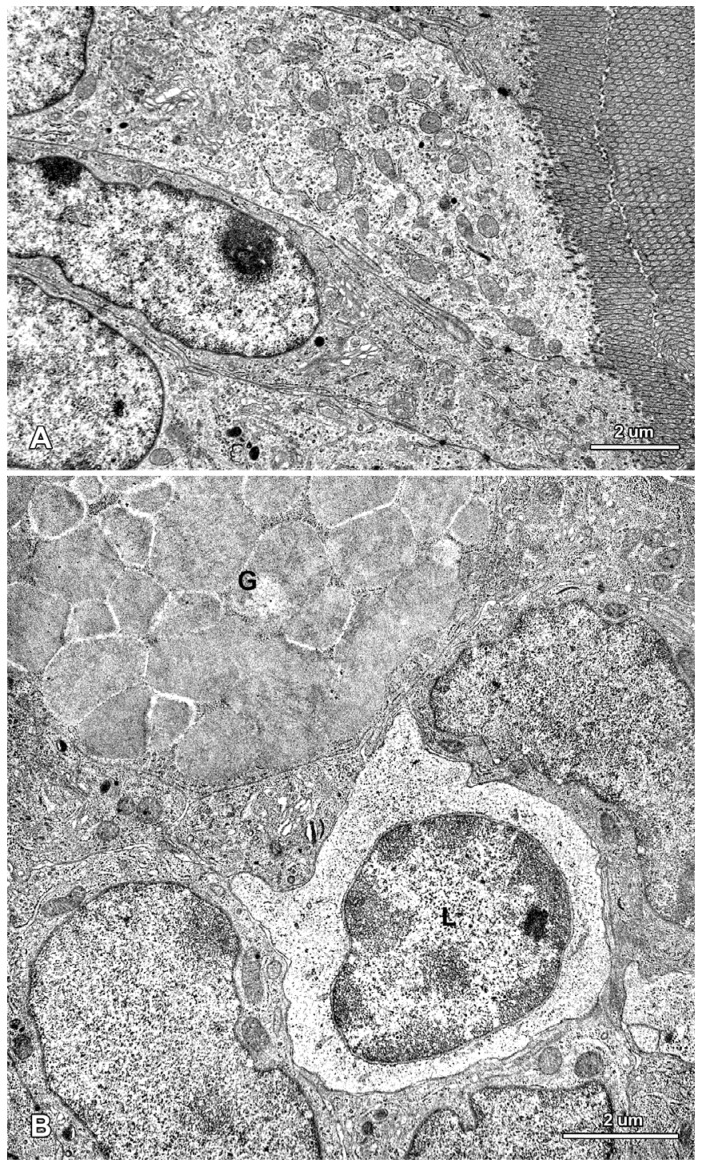
Ultrastructure of the jejunal mucosa epithelium in a control pig after six weeks of the experiment. (**A**) Enterocytes with a characteristic structure of the apical part; (**B**) An intraepithelial lymphocyte (L) and a fragment of a goblet cell (G).

In the control pigs, the epithelium covering villi and forming crypts showed a typical organization in samples taken after one, three, and six weeks of the experiment. The absorptive cells were the major epithelial component and showed the polarity of cellular organization (Figure 5A). These cells were tall and columnar with a basally situated, oval nucleus. The basal part of the cytoplasm contained a few mitochondria and profiles of rough and smooth endoplasmic reticulum. In the apical part of the cytoplasm many mitochondria, elements of the endoplasmic reticulum, and microtubules were observed. The Golgi apparatus was located close to the nucleus. The apical surface formed well-developed microvilli. The adjoining cell membranes of the absorptive cells were close to each other, and the junctional complexes were well-formed. The goblet cells were interspersed among the absorptive cells and had a typical ultrastructure (Figure 5B). Lymphocytes were present between the epithelial cells (Figure 5B).

The lamina propria had a classical ultrastructure with abundant immunocompetent and migratory cells in all of the control pigs. The crypts of Lieberkühn were simply tubular with a typical cellular composition.

**Figure 6:**
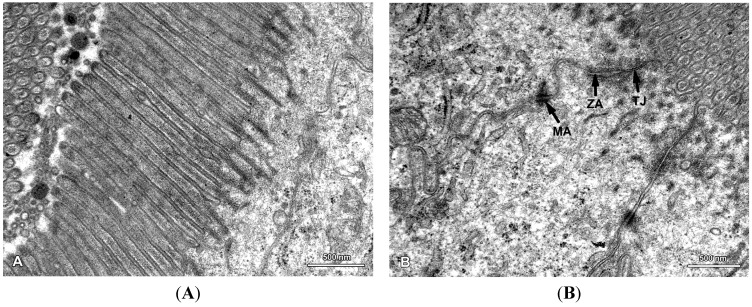
Ultrastructure of the jejunal mucosa epithelium in a pig that received DON for six weeks. (**A**) Regularly distributed, parallel microvilli on the apical surface of the adsorptive cells; (**B**) Junctional complexes between the adsorptive cells: TJ, tight junction; ZA, zonula adherens (belt desmosome); MA, macula adherens (spot desmosome).

**Figure 7:**
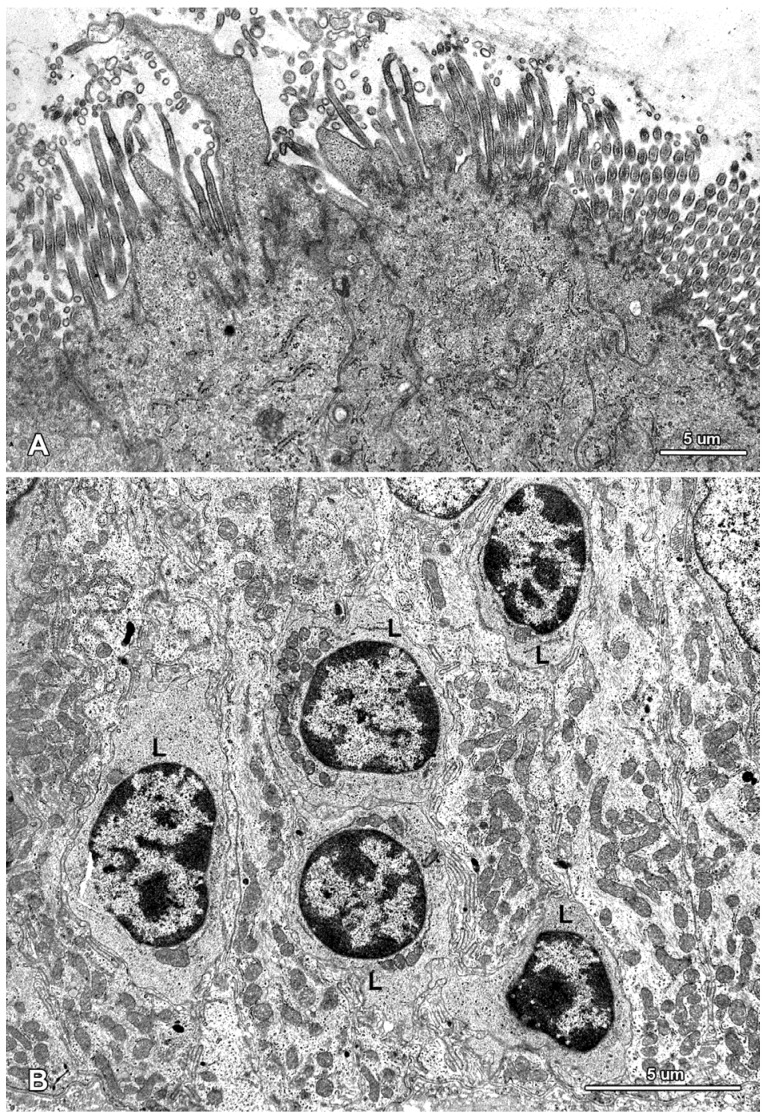
Ultrastructure of the jejunal mucosal epithelium in a pig that received ZEN for one week. (**A**) Drop-like protrusions extending from the apical surface of the enterocytes; (**B**) Lymphocytes (L) between epithelial cells.

**Figure 8:**
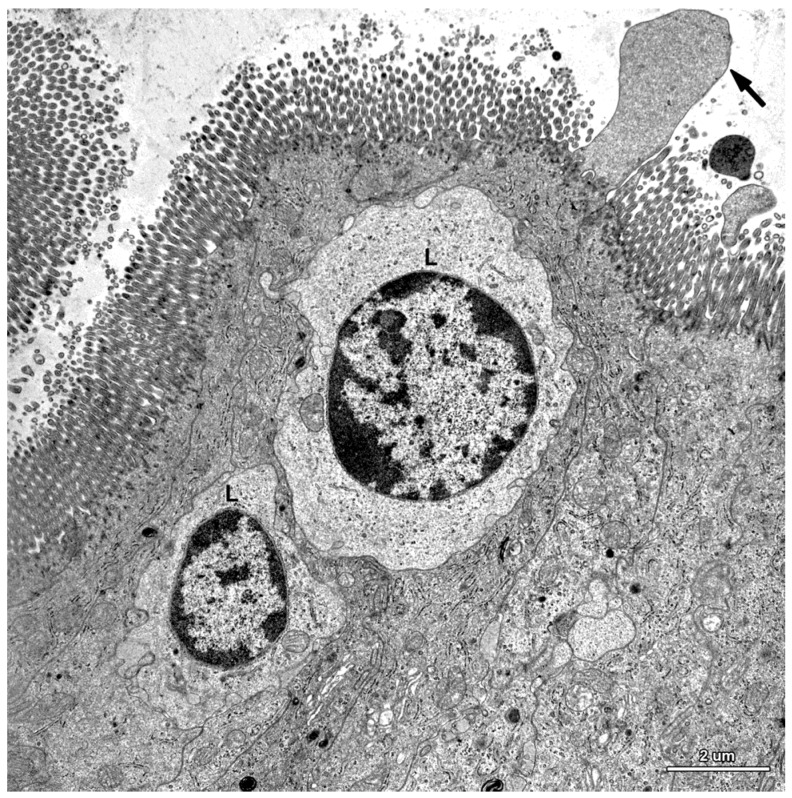
Ultrastructure of the epithelium in a pig that received DON + ZEN for six weeks. Note the drop-like protrusion (arrow) extending from the apical surface of enterocyte and numerous lymphocytes (L) between the epithelial cells.

**Figure 9:**
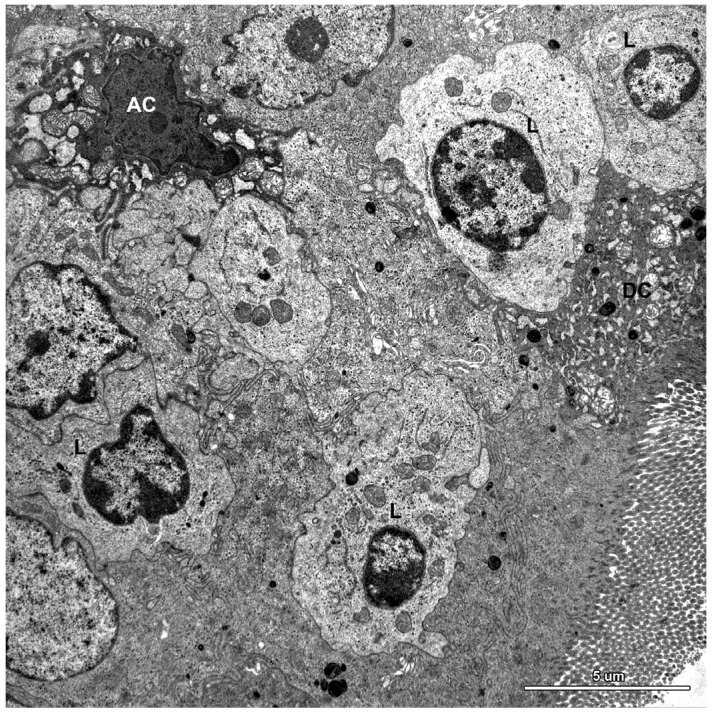
Ultrastructure of the jejunal mucosa epithelium in a pig that received DON for six weeks. Note numerous lymphocytes (L) between epithelial cells, degenerating adsorptive enterocytes (DC), and apoptotic cells (AC).

Treatment with ZEN, DON and DON + ZEN did not cause prominent pathological alternations in the epithelial cells forming villi and crypts; however, some differences in the ultrastructure between the experimental and control pigs were noted (Figure 6, Figure 7, Figure 8 and Figure 9).

In this study, we paid special attention to the microvilli of the absorptive cells because on one hand, they are exposed to the highest levels of toxin and on the other hand, they play a crucial role in nutrient adsorption. In the samples taken from the control and mycotoxin-treated pigs, well-developed, parallel microvilli covered the entire apical surface of the enterocytes (Figure 6A). In the pigs receiving ZEN and DON + ZEN, adsorptive cells with drop-like protrusions of apical cytoplasm that affected the distribution of the microvilli were also noted (Figure 7A and Figure 8). These cells occurred infrequently but were found in all gilts treated with ZEN.

The next facet of our special interest included the cell junctions between epithelial cells because previous studies reported that DON can affect the intestinal barrier [14,17]. In all groups of pigs, the junctional complexes were well-developed and comprised tight junctions, belt desmosomes, and spot desmosomes (Figure 6B). Therefore, it could be concluded that the low doses of mycotoxins used in our study did not affect the ultrastructure of the cell junctions.

The administered mycotoxins also did not change the internal organization of the epithelial cells and the morphology of organelles, including the mitochondria, the smooth and granular endoplasmic reticulum, and the Golgi apparatus (Figure 5, Figure 7, Figure 8 and Figure 9).

A characteristic feature of the epithelium covering the villi was the presence of numerous lymphocytes in samples taken from pigs that received ZEN for one week as well as ZEN, DON or DON + ZEN for three and six weeks (Figure 7, Figure 8 and Figure 9).

Some degenerating cells and apoptotic cells were found in the epithelium in all animals (Figure 9); however, there were no difference in the frequency of their occurrence between groups.

**Figure 10:**
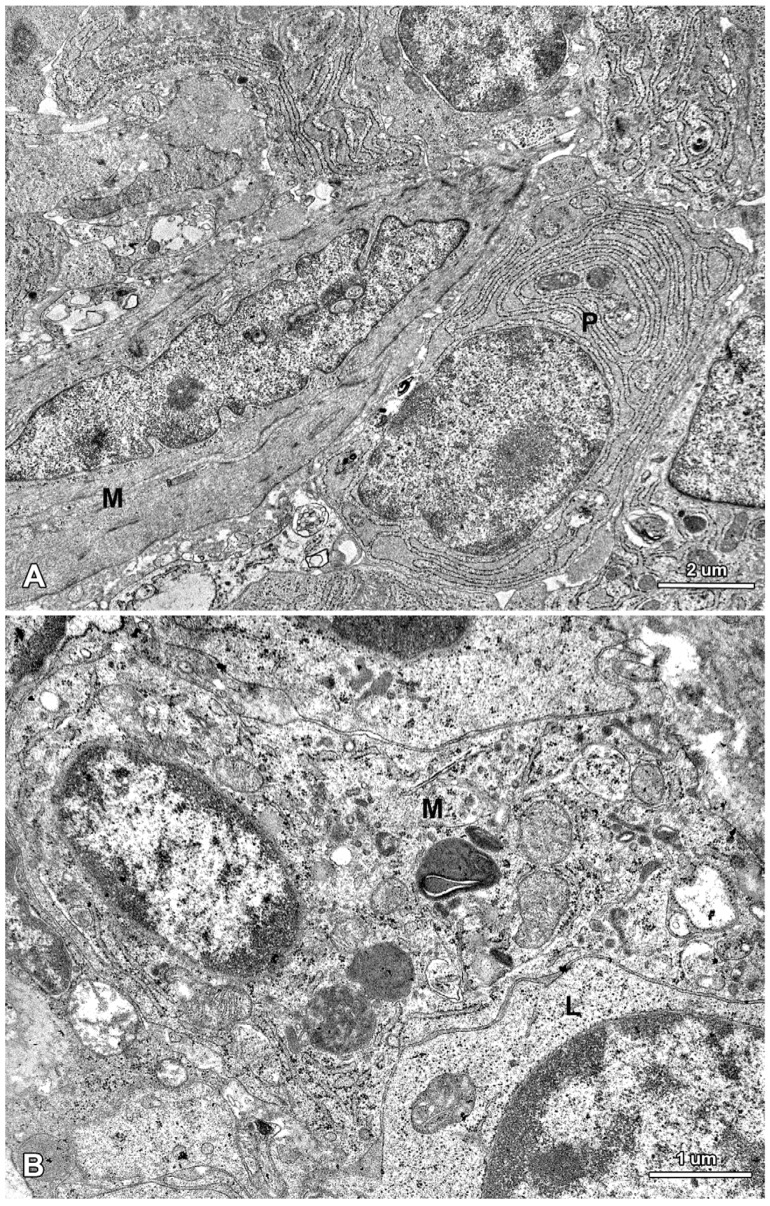
Ultrastructure of the jejunal mucosa lamina propria in a pig that received DON for six weeks. (**A**) Plasma cells (P) and myocyte (M) in the villus; (**B**) Macrophage (M) and lymphocyte (L) in the stroma between the intestinal crypts.

The lamina propria of the jejunum of pigs treated with mycotoxins was formed by a loose connective tissue with numerous cells: fibrocytes and fibroblasts, lymphocytes, plasma cells, mast cells, eosinophils, and macrophages (Figure 10). Myocytes were observed inside of the villi (Figure 10). The difference between the groups was due to plasma cells, which were more numerous in the lamina propria of gilts treated with ZEN or DON for one, three, and six weeks than in the control pigs.

In contrast to the histological data, the results of ultrastructural studies dealing with the effect of *Fusarium* mycotoxins on the intestine have been infrequently published [12,42]. Therefore, a comparative analysis of the results is impossible. As in the present study, no negative effects on the subcellular structure of the jejunal epithelium were reported after treatment of gilts with ZEN at doses of 200 and 400 μg/kg BW for seven days [42]. In the *in vitro* studies, incubation of jejunal explants with 10 μM DON resulted in an increase in intercellular spaces, a decrease in the size and number of microvilli, and a loss of junction complexes [12].

### 2.3. Functional Analysis of Epithelial Cell Junctions Using Lanthanum Ions

**Figure 11:**
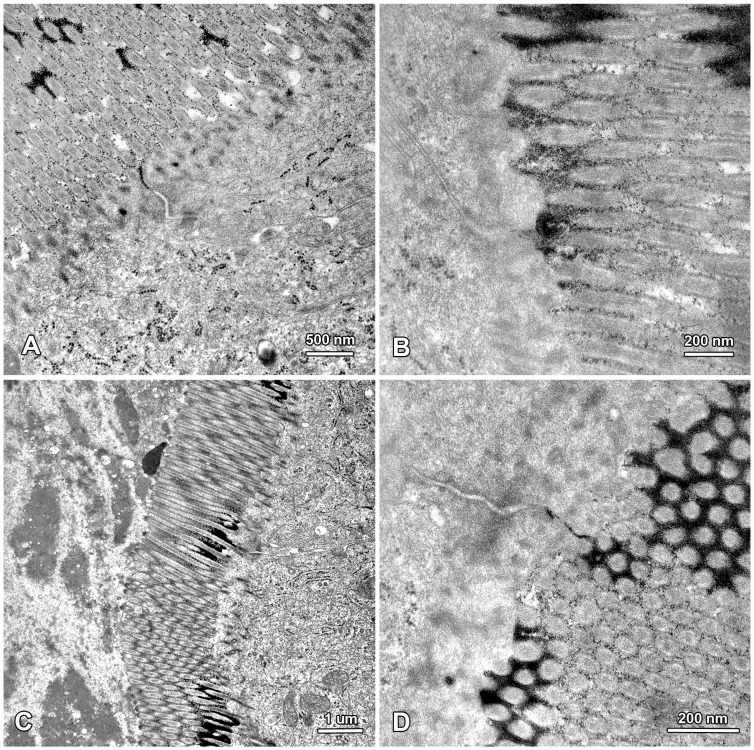
The apical parts of enterocytes in samples fixed using lanthanum nitrate. Note that no deposits are present in the intercellular spaces below the tight junctions. (**A**,**B**) The jejunum of a control pig fixed after six weeks of the experiment; (**C**) The jejunum of a pig treated with DON for six weeks; (**D**) The jejunum of a pig treated with ZEN for six weeks.

In order to check the effect of mycotoxins on permeability of the epithelial barrier, the samples were fixed using the lanthanum technique, which is the gold standard in the studies of tight junctions at an ultrastructural level [57,58,59]. In all of the samples, the electron dense lanthanum particles were observed between the microvilli, on the surface of cell membrane and in the most apical parts of the intercellular spaces above the tight junctions (Figure 11). However, no deposits were present in the intercellular spaces below the tight junctions (Figure 11); therefore, it could be concluded that DON and ZEN did not affect the permeability of the jejunal epithelium at the doses used. It should be noted that a proposed mechanism for DON action includes a reduction in claudin and occludin expression [12,13].

### 2.4. Summary

The treatment of immature gilts with low doses of ZEN and DON induced changes in the jejunum morphology, which were dependent on the type of mycotoxin, the manner of administration (separately or in combination), and the duration of exposure. The effects of ZEN were observed during the initial period of experiment, and they were partially reduced in the successive weeks of exposure. In general, ZEN had a prominent effect on the local immune system, leading to an increase in the number of lymphocytes in the villus epithelium and plasma cells in the lamina propria. It also induced a transient increase in the percentage of goblet cells in the villus epithelium. ZEN did not affect the mucosal architecture. Moreover, an electron microscopy study failed to show an adverse effect of ZEN on cells located in the epithelium and the lamina propria, except for a sporadically observed formation of drop-like protrusions on the apical surface of the enterocytes. DON also affected the jejunum morphology, but unlike ZEN, its effects were mainly manifested after prolonged administration. The six-week-long treatment with DON led to an increase in the mucosal thickness and the depth of the intestinal crypts. Similarly to ZEN, the changes induced by DON mostly concerned the local immune system and included an increase in the number of lymphocytes in the villus epithelium and lymphocytes and plasma cells in the lamina propria. Electron microcopy demonstrated that the dose of DON used in this study did not cause any changes in the ultrastructure of the mucosal epithelium and the lamina propria nor disturb the intestinal barrier. The results of the joint administration of both mycotoxins indicated complex interactions between them.

Based on the animal weight and the average feed intake, it could be estimated that the dose of DON used in this study corresponds to a toxin feed concentration of approximately 250 µg per kg, which is much lower than the limit (0.9 mg/kg of feed) recommended as acceptable for complementary and complete feedstuffs for pigs by the European Commission [36]. In view of our results, even very low levels of DON, below the recommended limit and the NOAEL, could have an effect on the jejunal mucosa, especially the local immune system, and be potentially hazardous. A study by Alizadeh *et al.* [48] published in 2015 showed that administration of feed containing DON at 0.9 mg/kg of feed for as little as 10 days induced adverse histological and biochemical changes in pig intestine. The results of this study indicate that the issue of safe and toxic levels of DON is still unresolved.

## 3. Experimental Section

### 3.1. Animals and Experimental Design

The study was performed on 36 clinically healthy mixed breed gilts (White Polish Big × Polish White Earhanging), with an initial body weight of 25 ± 2 kg, purchased from a farm on which they received feed without detectable amounts of ZEN, α-ZEL or DON. Tests for the presence of mycotoxins in the feed were performed as described previously [60]. Serological tests were carried out to exclude Auyeski’s disease, mycoplasmosis, parvovirosis, actinobacillosis, and porcine reproductive-respiratory syndrome in these animals. The tests for internal and external parasites also gave negative results. The pigs were fed twice daily and had free access to water. The diet composition was presented in Table 1.

**Table 1:** Composition of the diet.

Ingredients	%	Composition	g/kg
Wheat middlings	60.2	Starch	449.0
Barley middlings	20.0	Crude protein	204.7
Post-extraction soya meal	16.0	Crude fiber	49.3
Chalk	0.5	Fat	32.3
Multipremix PWT/3E	3.0	Ash	82.2
Zitrosan	0.3	-	-

The animals were divided into three experimental groups (Z, D, and M; *n* = 9 in each group) and a control group (C; *n* = 9). The animals in group Z received ZEN at a dose of 40 μg/kg BW per day, the animals in group D received DON at a dose of 12 μg/kg BW per day, and the animals in group M received a mixture of ZEN and DON (40 μg ZEN/kg BW + 12 μg DON/kg BW per day). The mycotoxins were synthesized and standardized at the Department of Chemistry, Faculty of Wood Technology, Poznan University of Life Sciences, Poland. The mycotoxins were administered orally during the morning feeding in water-soluble capsules containing oat bran as a vehicle. The gilts were weighed each week to establish the amount of DON and ZEN given to each animal. The animals in group C received capsules without mycotoxins.

Three animals from each experimental group were killed by intravenous administration of sodium pentobarbital (Vetbutal, Biowet, Puławy, Poland) and exsanguination after one, three, and six weeks after the beginning of the experiment. The tissue samples were taken from the middle part of the jejunum within 3 min after cardiac arrest.

All procedures were carried out in compliance with Polish legal regulations determining the terms and methods for performing experiments on animals and the European Community Directive for the ethical use of experimental animals. The protocol was approved by the Local Ethical Council in Olsztyn (opinion No. 88/N of 16 December 2009).

### 3.2. Histological Examinations

Tissue samples (two per each animal) were fixed in 4% paraformaldehyde in phosphate buffer for 48 h and embedded in paraffin. The 4-μm-thick sections were stained using the hematoxylin and eosin method (HE), the periodic acid Schiff method (PAS), and the methyl green-pyronine method (MGP) using an automated multistainer ST 5020 (Leica, Wetzlar, Germany). The slides were labelled so that those who performed the microscopic analysis did not know the type and duration of the treatment. The specimens were analyzed and photographed in an Axioimager light microscope equipped with an AxioCam MRc5 camera (Carl Zeiss, Oberkochen, Germany).

For morphometrical evaluation, the sections were scanned in a Mirax Desk scanner (Carl Zeiss). The following parameters were determined: the thickness of mucosa, the length of villi, the crypt depth, the ratio of villus height to crypt depth, the percentage of goblet cells in epithelium covering villi and forming intestinal glands, the number of lymphocytes per 50 epithelial cells in the villus epithelium, the number of lymphocytes and plasma cells per 10,000 μm^2^ of the lamina propria. Goblet cells were counted in PAS-stained sections and plasma cells in MPG stained sections. All other measurements were performed in HE-stained sections. The linear measurements were repeated 20 times per animal, the percentages of lymphocytes and goblet cells were determined by counting their number per 50 epithelial cells in 10 randomly selected areas, and the density of lymphocytes and plasma cells in the lamina propria by counting the cells in 10 randomly selected areas of 6000 to 12,000 µm^2^ each. The measurements were performed using Pannoramic Viewer 1.15 software (3D-Histech, Budapest, Hungary) and AxioVision 4.8 software (Carl Zeiss).

### 3.3. Ultrastructural Examinations

Jejunal mucosal samples were immersion-fixed in a mixture of 1% paraformaldehyde and 2.5% glutaraldehyde in 0.2 M phosphate buffer (pH 7.4) for 2 h at 4 °C, washed and post-fixed in 2% osmium tetroxide for 2 h. After dehydration, the samples were embedded in Epon 812. Semi-thin sections were cut from each block of tissue, stained with 1% toluidine blue, and examined under a light microscope in order to choose the sites for preparation of ultrathin sections. Ultrathin sections, contrasted with uranyl acetate and lead citrate, were examined with a Tecnai 12 Spirit G2 BioTwin transmission electron microscope (FEI, Hillsboro, OR, USA) equipped with two digital cameras: Veleta (Olympus Soft Imaging Solutions, Münster, Germany) and Eage 4 k (FEI).

### 3.4. Lanthanum Procedure

The mucosal specimens were fixed for 2 h in a freshly prepared mixture of 2.5% glutaraldehyde and 1% lanthanum nitrate, La(NO_3_)·6H_2_O, in cacodylate buffer (pH 7.2). Next, the samples were rinsed for 30 min in the cacodylate buffer containing 1% lanthanum nitrate and postfixed for 2 h in a freshly prepared mixture of 1% osmium tetroxide and 1% of lanthanum nitrate in cacodylate buffer. Then, the tissues were dehydrated and embedded in Epon 812. Unstained, ultrathin sections were examined with a Tecnai 12 Spirit G2 BioTwin transmission electron microscope (FEI).

### 3.5. Statistical Analysis

The data were analyzed using two-way ANOVA with Duncan’s test as a *post hoc* procedure. Statistical analyses were performed using Statistica 10.0 software (StatSoft, Tulsa, OK, USA).

## 4. Conclusions

The obtained data show that low doses of ZEN and DON have no or a very weak effect on the epithelial cells covering the jejunal mucosa. However, these toxins significantly influence the intestinal immune system and may contribute to the development of subclinical inflammation and allergy. The possibility that very low doses of *Fusarium* mycotoxins along with other exogenous factors such as bacterial antigens play an important role in the control of homeostasis of the intestinal local immune system should also be considered.
